# Functional significance of opioid receptor homomers and heteromers

**DOI:** 10.1038/s41467-025-64693-4

**Published:** 2025-11-07

**Authors:** Sergi Ferré, Francisco Ciruela, Leonardo Pardo

**Affiliations:** 1 Integrative Neurobiology Section, National Institute on Drug Abuse, Intramural Research Program, National Institutes of Health, Baltimore, MD USA; 2 Pharmacology Unit, Department of Pathology and Experimental Therapeutics, School of Medicine and Health Sciences, Institute of Neurosciences, IDIBELL, University of Barcelona, L’Hospitalet de Llobregat, Spain; 3 Laboratory of Computational Medicine, Biostatistics Unit, Faculty of Medicine, Autonomous University of Barcelona, Bellaterra, Spain

**Keywords:** Biophysical chemistry, Neurochemistry

## Abstract

A new structural dynamics analysis provides an explanatory bridge between apparently divergent results in the field of opioid receptor oligomerization and, by extension, in the broader field of class A G protein-coupled receptor oligomerization.

It has been established that G protein-coupled receptor (GPCR) oligomerization is associated with functional changes, and as such homomers and heteromers with functional and pharmacological properties different from their respective monomers present as novel targets for the development of new selective therapeutics^1–3^.

GPCR oligomerization has been a matter of intense debate especially for class A GPCRs, which constitutes the largest family of GPCRs and includes the three subtypes of opioid receptors (ORs), µ, δ and κ (MOR, DOR and KOR)^2,3^. Although there is less reluctance to accept GPCR oligomers’ presence in transfected cells, the debate is now centered on their existence in native tissues, their real physiological density, and their putative functional and pharmacological significance in vivo^2,3^.

Due to the high number of G protein-coupled receptors (GPCRs), roughly ~800 (~350 for non-sensory GPCRs), the probability for casual, non-functional, heteromer formation is very high. However, there are few examples that fulfill the proposed three criteria for functional GPCR heteromerization: i) colocalization and interaction, ii) distinct oligomer-specific properties, and iii) concomitant disruption of these properties with specific oligomer-disruptive tools^3^. OR heteromers fulfilling these criteria include: the MOR-DOR heteromer, which is involved in the analgesic effects of opioids^3–5^; the MOR-galanin Gal_1_ receptor (MOR-Gal_1_R) heteromer, involved in the dopaminergic and euphoric effects^6,7^; and the MOR-corticotropin releasing factor CRF_1_ receptor (MOR-CRF_1_R) heteromer, which appears to be involved in the antistressor and antidepressant effects^8^.

Initial evidence for the possible existence of class A GPCR homomers and heteromers was provided by in vitro proximity-based biophysical techniques, including bioluminescence and fluorescence resonance energy transfer and bimolecular complementation, using heterologous expression systems^2,3^. These techniques measure the close proximity of receptors fused to different chromophores, chromophore-activating enzymes, or their complementary moieties, and they require a purportedly high receptor density, all of which raises questions about their physiological relevance. Nevertheless, multiple studies documented distinct biochemical properties, “biochemical fingerprints”, of GPCR heteromers in recombinant systems, which was often used as a heuristic for the presence of heteromers in the native tissue^1–3^. However, the unequivocal demonstration of endogenous GPCR oligomerization requires the physical disruption of the oligomer in native tissues using oligomer-specific disruptive tools, together with the concomitant loss of its putative emergent functional properties. Prominent tools include synthetic peptides that directly compete with the oligomer interface by mimicking the amino acid sequence of transmembrane domains (TMs) and intra- or extracellular domains that form part of the interface, and small molecules that specifically target the amino acid residues involved in oligomerization^2–4,9^.

Several recent studies using single fluorescent-molecule imaging techniques have reported contrasting results about OR homodimerization^10–13^. An important limitation of these techniques is that visualization and tracking of the fluorescent molecules require a relative low density of receptor density (typically below 1 protomer/µm^2^). A previous study using this methodology to analyze homomerization of different GPCRs showed that the proportion of protomers existing as homodimers increases as the protomer density increases^14^. Nevertheless, the new results from single-molecule imaging experiments by Zhou et al. shed light on this debate, demonstrating MOR, DOR and KOR homodimerization, as well as MOR-DOR and DOR-KOR heterodimerization at relative low densities (around 1 protomer/µm^2^)^15,16^. Using single fluorescent-molecule tracking with total internal reflection fluorescence microscopy (TIRF), the authors demonstrated that transfected ORs, labeled with two different fluorescent colors, often exhibited transient colocalization and co-diffusion. This observation highlights the frequent formation of transient, metastable, homodimers and heterodimers in the plasma membrane. As an extension of their previous work with another GPCR, the chemoattractant N-formyl peptide receptor^17^, they developed a simplified methodology and robust algorithms that provide an accurate estimate of the kinetic constants behind the dynamics of GPCR homo and heterodimerization. First, the GPCR dimer dissociation rate constant or k_off_, which represents the inverse of the homodimer or heterodimer lifetime; second, the GPCR dimer-monomer dissociation equilibrium constants (K_D_), which represents the density of molecules (in number of protomers per µm^2^) present in the membrane when 50% of the protomers are forming homodimers or heterodimers; and third, the GPCR dimer association rate constant or k_on_, calculated as K_off_/K_D_ (Fig. 1). Then, since these parameters are independent of the receptor density, they can be used to easily calculate the percentage of protomers existing as homodimers or heterodimers at different plasma membrane receptor densities.

**Fig. 1:**
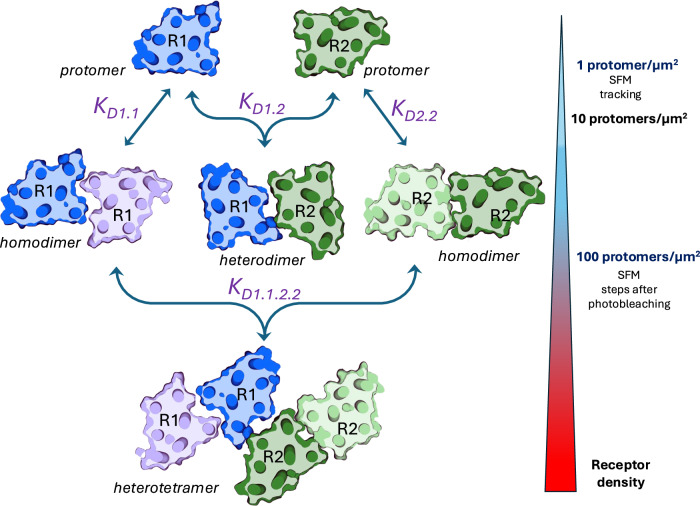
Scheme of the dynamics of oligomerization of two different GPCRs, R1 and R2, and their ability of forming homodimers, heterodimers and heterotetramers. *K*_*D1.1*_, *K*_*D1.2*_ and *K*_*D2.2*_ represent the GPCR dimer-monomer dissociation equilibrium constants, the density of molecules (in number of protomers per µm^2^) present in the membrane when 50% of the protomers are forming homodimers or heterodimers. These constants are independent of the GPCR expression levels and can be determined from single-fluorescent molecule (SFM) tracking analysis, which requires densities of transfected receptors ≤1 protomer/µm^2^. *K*_*D1.1.2.2*_ represents the hypothetical tetramer-dimer dissociation equilibrium constant, at higher densities of transfected receptors (putatively ≥100 protomers/µm^2^), which can be indirectly calculated from SFM experiments analyzing the number of steps after photobleaching. The proportion of protomers existing as homodimers or heterodimers in native tissues can be established when knowing the real plasma membrane receptor densities.

The studies by Zhou et al. underscore the critical importance of evaluating the homodimer-monomer and heterodimer-monomer dissociation equilibrium constant K_D_, rather than simply trying to detect homodimers or heterodimers. The authors first firmly establish that at relatively low densities the different OR subtypes form transient metastable homodimers and heterodimers with short (subsecond) lifetimes. The lifetimes of MOR-DOR and DOR-KOR heterodimers are approximately 250 ms, or roughly twice as long as those of the homodimers (120 ms for DOR-DOR and MOR-MOR and 180 ms for KOR-KOR homodimers)^15,16^. Although the oligomers have short lifetimes, the newly dissociated monomers quickly form new homodimers and heterodimers either with the same or different partner protomers. Furthermore, this occurs more easily as receptor densities increase. Based on the kinetic constants, their analysis indicates that OR monomers are more prevalent at densities of around 1 protomer/µm^2^, but that the proportion of protomers forming OR homomers or heteromers predominate at densities higher than 10 protomers**/**µm^2^. Their analysis also indicates that more complex oligomeric structures, such as trimers, can also form at these relative higher densities^15^. Altogether, these results indicate that substantial amounts of OR homomers and heteromers should exist in various nerve tissues at any time^15,16^.

Apart from the evidence of the existence of interactions between ORs, the studies by Zhou et al. fulfilled the other two criteria of GPCR oligomerization. Importantly, they show that at relatively low densities, the relative short lifetime of OR oligomers is sufficient to promote signaling distinct from OR monomers^15,16^. This included an increased signaling and internalization of the MOR by the transient metastable MOR-DOR heteromers^15^, in agreement with previous experiments using higher OR densities^3–5^. The third criterion was fulfilled by demonstrating the specific disrupting effects of oligomer-disrupting peptides^15,16^. Finally, in agreement with previous studies^3–5^, MOR-DOR heteromers were shown to be involved in the tolerance to the analgesic effects of morphine in experiments with in vivo intracerebroventricular administration of a heteromer-disrupting peptide^16^.

The critical, yet unanswered question in the evaluation of the physiological role of GPCR oligomers (and therefore of their functional significance) is the physiological, in vivo density of GPCR protomers within specific plasma membrane domains of specific cells, such as in the dendrites, dendritic specializations or nerve terminals. Therefore, although necessary to understand the dynamics of GPCR oligomerization, the use of single fluorescent-molecule imaging techniques that require relatively low receptor densities can fall short at elucidating the different and predominant functional GPCR oligomeric structures. Determining the number of steps after photobleaching is another method that uses single fluorescent-molecule imaging/TIRF to elucidate the stoichiometry of GPCR oligomers in cells expressing higher protomer densities. As expected, using this technique, we have recently shown that under these conditions MOR dimers predominate over MOR monomers, in experiments with cells only transfected with fluorescent MORs and with cells co-transfected with Gal_1_Rs or with CRF_1_Rs fused to a different fluorescent molecule^7,8^. The analysis of co-localized fluorescent receptors allowed the frequent identification of tetrameric structures, of MOR-Gal_1_R or MOR-CRF_1_R heterotetramers composed of MOR and Ga_1_R or CRF_1_R homodimers^7,8^. The use of heteromer-disruptive peptides allowed to uncover the different homomeric and heteromeric interfaces as well as to determine the distinctive biochemical properties of the heteromer^7,8^.

The studies by Zhou et al. provide a bridge between the previous apparently divergent results in the field of GPCR oligomerization, where existence of class A GPCRs and specially OR oligomers was debated. Their dynamic analysis shows that ORs form metastable oligomers even with low OR densities with demonstrable oligomer-specific functional properties. By establishing the constants that determine the dynamics of OR homo and heteromerization, the percentage of ORs oligomers can then be estimated for any receptor density. The next important challenges in the field become: first, the determination of the actual physiological density of the possibly interacting GPCRs in the specific neuronal domains under study; and second, the analysis of the dynamic constants of GPCR heterotetramers and other possible GPCR oligomers that only become evident at higher yet most probable physiological densities.
